# Supplementary material for: Thermodynamics and Kinetics of Glycolytic Reactions. Part I: Kinetic Modeling Based on Irreversible Thermodynamics and Validation by Calorimetry
Source: Int J Mol Sci. 2020 Nov 6;21(21):8341. doi: 10.3390/ijms21218341 (PMC7664384; doi:10.3390/ijms21218341)
Supplement: Supplementary file 1 [file ijms-21-08341-s001.pdf]

Supplementary Material

Thermodynamics and Kinetics of Glycolytic Reactions. Part I: Kinetic Modeling Based on Irreversible Thermodynamics and Validation by Calorimetry

Kristina Vogel [1,2], Thorsten Greinert [3], Monique Reichard [1], Christoph Held [3], Hauke Harms [1] and Thomas Maskow [1,*]

[1] UFZ–Helmholtz Centre for Environmental Research, Department of Environmental Microbiology, Leipzig, Permoserstr. 15, D-04318 Leipzig, Germany; Kristina.vogel@ufz.de (K.V.); monique.reichard@ufz.de (M.R.); Hauke.harms@ufz.de (H.H.)

[2] Institute for Drug Development, Leipzig University Medical School, Leipzig University, Bruederstr. 34, 04103 Leipzig, Germany

[3] Laboratory of Thermodynamics, Department of Biochemical and Chemical Engineering, Technische Universitaet Dortmund, Emil-Figge-Str. 70, 44227 Dortmund, Germany; thorsten.greinert@tu-dortmund.de (T.G.); christoph.held@tu-dortmund.de (C.H.)

* Correspondence: thomas.maskow@ufz.de

Contents

1. Chemicals used in this work 2

2. ePC-SAFT 2

3. Statistics 4

4. References 5

1. Chemicals used in this work

Table S1. Chemicals used in this work.

Substance	CAS-number	M_i / $g \cdot mol^{-1}$	Supplier	Purity (%)
Enolase (*Saccharomyces cerevisiae*)	9014-08-8	93,069	Sigma Aldrich	-
Phospho(enol)pyruvic acid monosodium salt hydrate	53823-68-0	190.02	Sigma Aldrich	98.9
Phosphoglucose isomerase type III from baker's yeast	9001-41-6	119,500	Sigma-Aldrich	-
Fructose 6 – phosphate disodium salt	26177-86-6	304.00	Alfa Aesar	95
MOPS	1132-61-2	209.27	AppliChem	≥ 99.5
Magnesium chloride hexahydrate	7791-18-6	203.3	AppliChem	-
Sodium chloride	7647-14-5	58. 4	CHEMSOLUTE	≥ 99
Sodium hydroxide	1310-73-2	40.00	Roth	≥ 98
HEPES	7365-45-9	238.31	Roth	≥ 99.5
Dipotassium hydrogen phosphate	7758-11-4	174.18	Roth	≥ 99
Potassium dihydrogen phosphate	7778-77-0	136.09	Roth	≥ 99

Purities are given in percent by mass. Suppliers are: Sigma Aldrich (Sigma-Aldrich Chemie GmbH, Steinheim, Germany), Roth (Bernd Kraft, Duisburg, Germany), AppliChem (AppliChem GmbH, Darmstadt, Germany), CHEMSOLUTE (Th. Geyer GmbH & Co. KG,Renningen, Germany), Alfa Aesar (Thermo Fisher (Kandel) GmbH, Kandel, Germany).

2. ePC-SAFT

Activity coefficients were predicted with the equation of state ePC-SAFT. ePC-SAFT as proposed by Held et al. [1] is based on the original version of PC-SAFT from Gross and Sadowski [2]. It is a revised version from ePC-SAFT developed by Cameretti et al. [3] and allows accounting for interactions between anions and cations which are present in the reaction medium.
The prediction of activity coefficients or other thermodynamic properties with ePC-SAFT is based on the calculation of the residual Helmholtz energy A^{res} from different contributions that are shown in eq. S1.

$$A^{res} = A^{hc} + A^{disp} + A^{assoc} + A^{ion} \qquad \text{(S1)}$$

The first of the four contributions is the Helmholtz energy A^{hc} of the hard-chain fluid, which is the reference fluid. The reference hard-chain system is itself composed of hard spheres. The other contributions account for perturbations to this reference fluid. The second contribution A^{disp} includes molecular dispersive interactions related to van der Waals forces. The third contribution A^{assoc} includes associative interactions related to hydrogen bonding forces. The fourth contribution A^{ion} includes ionic interactions and is described by a Debye-Hückel expression. The calculation of these four contributions with ePC-SAFT requires pure-component parameters and optionally binary parameters. Two of the pure-component parameters are the segment number m_i^{seg} and the segment diameter σ_i that describe the volume of the hard-chain reference system. Furthermore, the dispersion-energy parameter u_i/k_B including the Boltzmann constant k_B describes the dispersive interactions considered in A^{disp}. The hydrogen-bonding forces considered in A^{assoc} are described by the association-energy parameter $\varepsilon^{A_iB_i}/k_B$ and the association-volume parameter $\kappa^{A_iB_i}$. Last, the number of association sites N_i^{assoc} and the charge of an ion q are required.
Mixtures of substances are described using mixing rules to combine the pure-component parameters of the different substances. Lorentz-Berthelot [4] combining rules are used to determine the combined

segment diameter σ_{ij} and the combined dispersion-energy parameter u_{ij}/k_B of components i and j according to Eqs.S2 and S3.

$$\sigma_{ij} = \frac{1}{2}(\sigma_i + \sigma_j) \tag{S2}$$

$$u_{ij} = \sqrt{u_i u_j}(1 - k_{ij}) \tag{S3}$$

For mixtures, the binary interaction parameter k_{ij} is used to correct for deviations of u_{ij} from the geometric mean of u_i and u_j. Further, the association parameters $\varepsilon^{A_iB_j}$ and $\kappa^{A_iB_j}$ of two components i and j in a mixture were determined using the mixing rules proposed by Wolbach and Sandler [5].

$$\varepsilon^{A_iB_j} = \frac{1}{2}(\varepsilon^{A_iB_i} + \varepsilon^{A_jB_j}) \tag{S4}$$

$$\kappa^{A_iB_j} = \sqrt{\kappa^{A_iB_i}\kappa^{A_jB_j}}\left(\frac{\sqrt{\sigma_i\sigma_j}}{0.5(\sigma_i + \sigma_j)}\right)^3 \tag{S5}$$

Table S2. ePC-SAFT parameters applied in this work with the sources for the respective sets of parameters. For 2PG the parameters of its isomer 3PG were used.

	m_i^{seg}	σ_i	u_i/k_B	N_i^{assoc}	$\varepsilon^{A_iB_i}/k_B$	$\kappa^{A_iB_i}$	k_{i,H_2O}	z	Source
	-	Å	K	-	K	-	-	-	
PEP	12.007	2.200	407.3	2+2	5000	0.1	a	-2	[6]
2-PG	3.110	4.660	322.0	5+5	501.2	10^{-4}	b	-2	[7]
G6P	22.329	2.227	243.3	5+5	5000	0.1	-0.095	-	[8], TW(kij)*
F6P	35.594	1.810	198.5	5+5	5000	0.1	-0.255	-	[8], TW(kij)*
MOPS	15.697	2.271	171.6	2+2	4418	0.001	-0.150	-	[9]
HEPES	11.787	2.708	141.88	2+2	2645	0.043	-0.1798	0	TW**
$H_2PO_4^-$ c	1	3.651	95.0	-	-	-	0.25	-1	[1]
HPO_4^{2-} d	1	2.162	146.0	-	-	-	0.25	-2	[1]
water	1.205	e	353.9	1+1	2426	0.0451	-	-	[10]
Na^+ f	1	2.823	230.0	-	-	-	g	+1	[1]
Mg^{2+} h	1	3.133	1500	-	-	-	-0.25	+2	[1]
Cl^-	1	2.756	170.0	-	-	-	-0.25	-1	[1]

* TW(kij): the k_{ij} between $G6P^{2-}$ and $F6P^{2-}$ were refitted in this work to osmotic coefficient data from ref. [8] of water/$G6PK_2$ and water/$F6PK_2$ solutions, using the reference state infinite dilution in water. ** TW: parameters fitted in this work to osmotic coefficients and density data from Table S3.

a $k_{PEP,water}$ = -0.005083 T/K + 1.3316 [6]

b $k_{3-PG,water}$ = 0.002033 T/K - 0.7064 [7]

c $k_{H_2PO_4^-,Na^+}$ = -0.071 [8]

d $k_{HPO_4^{2-},Na^+}$ = -1.000 [9]

e σ_{water} = 2.7927 + 10.11 exp(-0.01775 T/K) - 1.417 exp(-0.01146 T/K) [10]

f k_{Na^+,Cl^-} = 0.3166 [11]

g $k_{Na^+,water}$= -0.007981 T/K + 2.3799 [12]

h k_{Mg^{2+},Cl^-} = 0.817 [13]

Osmotic coefficients and mixture densities of aqueous HEPES solutions at 25°C and 1 bar have been measured. Freezing-point osmometer FPO (Gonotec O10) and a vibrating U-tube densimeter (Anton-Paar DMA 4200) have been used for that purpose. All samples were prepared only once, and all measurements were carried out at least in triplicates. O10 apparatus was calibrated with water and a 300 mosm kg^{-1} standard NaCl solution. The DMA apparatus was calibrated with water and air at ambient pressure. The results are given in Table S3.

Table S3. Experimental osmotic coefficients ϕ of water+HEPES solutions obtained by FPO in this work and experimental densities ρ of aqueous HEPES solutions obtained by DMA 4200 at 25 °C in this work.

**Molality** m_{HEPES}	ϕ	**Mole Fraction** x_{HEPES}	ρ
(mol kg^{-1})	(-) at pH4	(-)	(kg $(m^3)^{-1}$)
0.1	1.00	0.000899	1000.756
1	1.09	0.001795	1004.281
1.4	1.20	0.005369	1018.052
		0.007141	1024.621
		0.008926	1031.396
		0.010666	1037.280
		0.017686	1059.576
		0.043089	1125.936
		0.067110	1167.464
		0.082408	1190.317

3. Statistics

Table S4. Statistical parameters of reaction 2 from Noor and flux-force models.

**Reaction 2**		**Noor**	**Flux-force**
**conditions**	replicates	R^2	R^2
298.15 K	1	0.99959	0.99855
	2	0.99957	0.99767
	3	0.99668	0.99894
305.15 K	1	0.99634	0.99935
	2	0.99909	0.99942
	3	0.95286	0.99911
310.15 K	1	0.99972	0.99921
	2	0.99968	0.99922
	3	0.99962	0.99904

Table S5. Statistical parameters of reaction 9 from Noor and flux-force models.

Reaction 9		Noor	Flux-force
conditions	replicates	R^2	R^2
298.15 K	1	0.98457	0.99459
	2	0.98378	0.99313
	3	0.99177	0.99659
305.15 K	1	0.99752	0.99722
	2	0.99945	0.99763
	3	0.99718	0.99792
310.15 K	1	0.99903	0.99892
	2	0.99953	0.9991
	3	0.99863	0.99883

4. References

1. Held, C.; Reschke, T.; Mohammad, S.; Luza, A.; Sadowski, G., ePC-SAFT revised. *Chemical Engineering Research and Design* **2014,** 92, (12), 2884-2897.
2. Gross, J.; Sadowski, G., Perturbed-chain SAFT: An equation of state based on a perturbation theory for chain molecules. *Industrial & engineering chemistry research* **2001,** 40, (4), 1244-1260.
3. Cameretti, L. F.; Sadowski, G.; Mollerup, J. M., Modeling of aqueous electrolyte solutions with perturbed-chain statistical associated fluid theory. *Industrial & Engineering Chemistry Research* **2005,** 44, (9), 3355-3362.
4. Lorentz, H., Ueber die Anwendung des Satzes vom Virial in der kinetischen Theorie der Gase. *Annalen der physik* **1881,** 248, (1), 127-136.
5. Wolbach, J. P.; Sandler, S. I., Using molecular orbital calculations to describe the phase behavior of hydrogen-bonding fluids. *Industrial & engineering chemistry research* **1997,** 36, (10), 4041-4051.
6. Greinert, T.; Vogel, K.; Seifert, A. I.; Siewert, R.; Andreeva, I. V.; Verevkin, S. P.; Maskow, T.; Sadowski, G.; Held, C., Standard Gibbs energy of metabolic reactions: V. Enolase reaction. *Biochimica et Biophysica Acta (BBA)-Proteins and Proteomics* **2020,** 1868, (4), 140365.
7. Wangler, A.; Schmidt, C.; Sadowski, G.; Held, C., Standard Gibbs energy of metabolic reactions: III the 3-phosphoglycerate kinase reaction. *ACS omega* **2018,** 3, (2), 1783-1790.
8. Hoffmann, P.; Held, C.; Maskow, T.; Sadowski, G., A thermodynamic investigation of the glucose-6-phosphate isomerization. *Biophysical chemistry* **2014,** 195, 22-31.
9. Vogel, K.; Greinert, T.; Harms, H.; Sadowski, G.; Held, C.; Maskow, T., Influence of cytosolic conditions on the reaction equilibrium and the reaction enthalpy of the enolase reaction accessed by calorimetry and van 't HOFF. *Biochimica et Biophysica Acta (BBA)-General Subjects* **2020,** 1864, (10), 129675.
10. Fuchs, D.; Fischer, J.; Tumakaka, F.; Sadowski, G., Solubility of amino acids: Influence of the pH value and the addition of alcoholic cosolvents on aqueous solubility. *Industrial & engineering chemistry research* **2006,** 45, (19), 6578-6584.
